# One Hundred Consecutive Cases of Skin Disease

**Published:** 1891-10

**Authors:** M. B. Hutchins

**Affiliations:** Lecturer on Diseases of the Skin, Atlanta Medical College, Atlanta, Ga.


### ONE HUNDRED CONSECUTIVE CASES OF SKIN DISEASE.*

### IX.

By M. B. HUTCHINS, M. D.,
Lecturer on Diseases of the Skin, Atlanta Medical College, Atlanta, Ga.

The case diagnosed as *xeroderma pigmentosum* was seen over a year ago, and this diagnosis was provisional. It is of sufficient interest to deserve separate publication, and I hope to be able to complete the history in such a manner as to make it worthy of being laid before my readers at some future day.

*ERRATUM.—In article VIII. "Carcinoma *Cuticulare*" should have been printed, Carcinoma *Lenticulare*.

### URTICARIA.

*Two Cases.*—First, a young lady of twenty-one. Began on arms six days before consultation. When seen, almost the entire skin surface was involved, regardless of localization. Lesions were " wheals " and papules, former average finger nail in size ; latter pea-sized ; the larger lesions of pinkish color, resembling mosquito bites. First symptom was itching. Rubbing the skin for relief of itching brought out the lesions. Scratching had caused the formation of a minute blood crust on top of papules, making these lesions, at a little distance, resemble those of scabies, but the hands were not affected.

Headache since beginning of eruption. Tongue slightly furred, patient of constipated habit. Appetite good.

*Treatment.*—Ordered to take a free dose of Epsom salts at bedtime. For future constipation the " cascara and nux " mixture—which has already been mentioned in these articles. Locally the following was ordered :

℞. Zinc ox., ʒvi.

Camphor., ʒiii.

Aquæ calcis., ʒiii.

Aquaæ, ad. ℥vi.

M. Sig.—Shake, and apply frequently.

This patient was not seen again. She got married three days later.

Second case, young man of 22. Whole cutaneous surface irritable and "wheals" easily caused to appear. On right wrist bright redness and two "wheals" the size of a silver half dollar. Redness of skin of lower third of thighs and about knees. Here and also on the legs were several red wheals from the diameter of a hazelnut to that of a half dollar, redness momentarily removable by pressure. Attack was preceded by irritability of skin and had been present for two days. A somewhat similar attack five weeks previous to present. Swelling of the face and upper lip was present, when patient first got up in the morning, in both attacks. Individual lesions of the disease remain only a few hours, but others continue to form. Patient thinks he had a similar disease in infancy. Severe attack of rheumatism five years preceding consultation, and occasional "touches" since. (Since this record was completed the gentleman has had another severe attack of rheumatism, lasting several weeks. Urticaria is not infrequently found in rheumatic subjects.)

*Treatment.*—Saline cathartic ordered. Spray face, if swells, with ether, and if upper lip again becomes swollen immerse it in hot water.

℞. Calamin. prep.,
Zn. ox., aa ℥ii.
Ether. sulph., ℥ii.
Hg. bichlor., gr. v.
Aquæ, ad. ℥vi.

M. Sig.—Shake and apply as often as possible.

Attack subsided within twelve hours. Cathartic was not taken and patient had a slight outbreak on posterior of thighs. Ordered to "open bowels freely" and continue treatment. Soon recovered.

### KERATOSIS PILARIS.

In a mild form this disease is not uncommon, but may exist in such a slight degree that the physician is not consulted about it. The case to be described was diagnosed as above. The patient was only seen for a moment, during his visit to the city. Later he wrote me a description of the trouble, and the following is taken chiefly from that letter:

Aged 28. On arms, abdomen and thighs, papules average pin-head to small shot in size. "Sometimes red." At times they contain in center, "a little yellow matter," "and when they dry up a little yellowish ball can be squeezed out, this sometimes containing a hair curled within it." Eruption not grouped save on one arm. After bathing there is itching on limbs, and general itching if he *does not* bathe. Itching severe on thighs in summer, after undressing. Itches after a bath until skin circulation is re-established. No itching after a Turkish bath. Family history: Half sister died of consumption; no other cases. Patient is a blonde and robust. Slight blepharitis marginalis.

*Treatment.*—Ordered bi-weekly bath in water of temperature of room, to each gallon of which half an ounce of cornstarch and two drachms of bicarbonate of soda were to be added, A. M. and P. M., and after bath to rub thoroughly with,

R. Ol. olivæ,

Lanolin., aa ℥i.

Saponis viridis, ℥vi.

M.

Internally he was to take emulsion of cod liver oil, beginning with teaspoonful after meals, and gradually increasing the dose to tablespoonful. The patient wrote rather disgustedly (*excusable*) of the treatment, but I encouraged him to keep it up, and two days later he wrote that it was "having a happier effect." This is the last record on the case.

POMPHOLYX.

*Two cases.*—First, gentleman of 29. Present about six years. Confined to feet, which sweat excessively and offensively. Constant formation of pin-head to pea-sized vesicles, between and "on" toes and on soles. After bathing and drying feet, marked itching. On inner side of left foot a round, defined, eczematous looking patch size of a silver dollar. General health robust. For "eczematous condition" was ordered

R. Ac. sal., gr. x.
Ungt. zn. ox., ℥i.

M. Sig.—Keep constantly applied.

To keep feet thickly covered with the following:

R. Resorcin pulv., ʒi-℈i.

Acid. bor., ʒii.

Amyli., ad. ℥iv.

M. Sig.—Dust well in socks.

Internally he received "Fowler's solution," two drops after meals, increasing dose at rate of one drop each succeeding day. Three days later, feet quite dry, itching better, one vesicle, eczematous condition improved. No further report.

Second case, gentleman of 45, with also seborrhoic eczema. Very large, deep bullæ flat, greenish looking, seated on toes and soles. Pricking permitted discharge of yellow, serous fluid. Itching at borders.

R. Resorcin pulv., ʒiv.

Zn. ox. pulv., ℥i.

Amyli., ℥ii.

M. Sig.—Keep well dusted on. Wash feet at night in water containing a little soda. No more blebs formed, and patient was still well at end of three weeks, when last seen.

(*Alopecia areata, etc., next issue, and conclusion.*)

1½ *Edgewood Ave.*

---

WHAT BECOMES OF DOCTORS AFTER GRADUATION.—In the *Medical Age*, Dr. W. R. Hubbert thus records the history of one hundred of his medical friends:

"I have endeavored to keep track of one hundred of my medical friends after graduation, especially of what they did during the first five years, and find nearly seventy-five per cent. had to resort to other employment to make a living. Twenty-three received a salary either in addition to practice or separate therefrom. Fifteen were proprietors of drug stores. Three were insurance agents. Four loaned money. One sold real estate. Three were connected with medical journals. One was an agent for drugs. One for books. One preached, One was in the patent medicine business. Two were farmers. One a manufacturer. Two gave massage treatment. One sawed wood, and subsequently suicided. Twelve gave up in disgust, and one never tried practice at all. Twenty-nine graduates only in one hundred exclusively devoted themselves to medicine, and of these, eleven associated themselves with other practitioners, and in many cases fell heir to their practice."

"In the western part of the county, that is, west of the Mississippi river, sixty per cent. of all physicians are connected with drug stores, either as clerks or proprietors. In the east the proportion is much less, being from twelve to fifteen per cent. In the west, also, forty per cent. of them have an interest in farms."

